# Medical and Philosophical Intelligence

**Published:** 1820-02

**Authors:** 


### Medical and Philosophical Intelligence.

WE have received a printed memoir, originally read to the New-York Historical Society, by Dr. Spalding, on the use of *scutellaria laterifolia*, (didynamia, ord. gymnospermia, Lin. dycotyledons, monopetales; ord. labiatæ, Jussieu,) as a means for the prevention and cure of *hydrophobia*. We must defer until the next Number a particular account of it; only now remarking, that its efficacy is better supported, and by respectable testimony, than any that has been heretofore proposed. The mode in which the *scullcap* appears to have been generally administered by the people who have most used it, is to infuse a tea-spoonful and a half of the dried herb, finely cut up, in a quart of warm water, and to drink half-a-pint of this infusion morning and night for two successive days; and on the third to omit it, and take a tea-spoonful of flowers of sulphur. This plan was pursued for forty days; during which time exercise was to be avoided, and an abstemious diet observed. The wound was dressed in a simple manner.

Dr. Fieve, in the *Gazette de Santé*, speaks in very high terms of the efficacy of the following embrocation for *chilblains*. He is convinced by very extensive experience, he says, that it is far superior to any other remedy in use. Take, Florence balsam, four ounces; muriatic acid, thirty drops; mix. The parts affected to be rubbed with it night and morning, until a good deal of heat is produced in them.

The Florence balsam is not noticed in our Pharmacopœia. CHAUMETON, in the *Dictionaire des Sciences Medicales,* says, "It contains a very great proportion of Venice turpentine, resin, &c. which furnish, on distillation, essential oils, which combine with alcohol. There passes first a limpid, pungent, spirituous liquor, in which the odour of turpentine is particularly distinguishable: this is the sort of essence termed *spirituous Florence balsam*, to distinguish it from that which passes in the subsequent period of distillation, which is a thickish oil of a citrine colour; this second product is called *oily Florence balsam*. The residue, partly carbonized, oily and watery, is known by the name of *black Florence balsam*. The first is almost the only sort now in use, &c. &c." Would not common *spirit of turpentine* answer the same purpose in the recipe of Dr. Fieve? This is in general use; but may not the muriatic acid be an important addition?

---

Mr. CURTIS, we have been informed, has adopted the practice of inflating the Eustachian tube, and injecting warm water into it, in some cases of deafness, with considerable advantage. The instrument he uses is an improvement of that of GUYOT, as described by GARENGEOT; the principal of which, we believe, is the employing tubes of caoutchouc instead of inelastic ones. Mr. Curtis considers that contraction of the Eustachian tube, or obstruction of it by mucous secretion, is not an unfrequent cause of deafness. He has not found the introduction of an elastic tube into the orifice of the Eustachian duct difficult in general; and, if the instrument be passed rather firmly along the parts in the introduction of it, it does not produce pain or other inconvenience by its irritation.

---

M. VAN MONS has made some interesting original observations, in his repetition of the experiments of Soemmering on the permeability of bladders and other animal membranes by water,* and the application of this property to the cold rectification of alcohol. The principal experiment is the following: A jug filled with alcohol was covered by some animal membrane, such as the amnios; bullocks', calves', or pigs', bladders; the swimming bladder of fishes. At the end of three months, the alcohol had lost from an ounce to an ounce and a half of its weight; but this loss consisted solely in the aqueous part of the fluid, for the liquid, which was of forty degrees strength, had acquired forty-four. This result was constant in a series of experiments with different membranes. He has found this discovery of Soemmering extremely favourable, either for the cold rectification of alcohol, the concentration of saline solutions and vegetable acids; and to various chemical operations, when it is necessary to separate water from any mixture which will not bear evaporation by fire.

---

* Published in the *Annales Générales des Sciences Physiques,* No. I. Bruxelles.

### *Brief Synopsis of the Philosophy of Material Phenomena, promulgated by* Sir Richard Phillips, 1817-18.*

1. The universe consists of extension of matter under various expansive, gazeous, fluid, and fixed, forms of body, proceeding in relative density from the rarest and most extended fluid media, to the most condensed aggregates of fixed atoms.

2. Body is susceptible of two varieties of motion: 1°, a motion or impulse of an aggregate, which occasions it to change its place in regard to other aggregates; and, 2°, a motion of the atoms of an aggregate, created when any impulse from any cause cannot produce commensurate change of place in the aggregate and diffuse the motion, so that, by re-action, the impulse terminates within the body in the mutual actions of its component atoms.

3. Motion of both kinds continues to affect a body, until it has been imparted or transferred to aggregates in contact, or has been diffused or radiated through the medium in which it is immersed; and this law of the equalization of motion, by the contact of moving aggregates and atoms with others susceptible of receiving and diffusing the motion, is the proximate cause of all varieties of material phenomena.

4. Motion appears, therefore, to constitute the life, power, and energy, of matter; and is the active soul of the universe. Matter is its patient, and the relative phenomena of bodies are the results. As it acts on aggregates by contact, or by impulse, on and through media, it constitutes the object of physical philosophy; and, as it affects compounds or structures of atoms, it is the object of chemical philosophy.

5. As no accident of matter can create motion, so all motion may be traced to some previously-existing motion, which has been transferred by mechanical combination; and, as existing motions are necessarily transferred and diffused, so no motion is lost; though, by its equal diffusion, it may cease to exhibit sensible phenomena.

6. The facility of receiving motion being equal to the facility of diffusing it, and motion in bodies constituting their power, all action and re-action are necessarily equal; and, motions being inversely as the number of atoms, all bodies act, therefore, on other bodies, at such distances as to produce equal momenta in the agent and patient: consequently, if free to move, or uninfluenced by paramount motions, their reciprocal actions and re-actions oblige them to revolve round a fulcrum or centre of the masses, necessarily producing equal momenta by forces of impulse, which, diverging through a sphere, are in divers bodies to each other inversely as the squares of their distances.

7. The motion of masses round a fulcrum or centre of masses, proves its mechanical origin, and that it is the effect of equilibrium of momenta, in which the masses, being constant quantities, the distances from the centre of motion must be inversely as those quantities.

8. Hence it is that the earth and moon revolve mechanically round the centres of their masses, at such distances, that their action and re-action on and through the medium of space, or their momenta, are equal; and, as the earth revolves at the same time round its own axis, and the centres of both these rotations do not accord; so librations of the movable fluids of the earth restore the balance, and occasion what are called tides of the sea and atmosphere, corresponding in direction and quantity with the varied positions of those two centres of terrestrial rotation, in relation of both to the sun or to the line of the earth's orbit. Thus, at the new and full moon, the distances are greatest from the orbit, and then the oscillations are greatest; and at the quarters they

* We insert this from its being a further application of the doctrine comprised in the "Essays" which we noticed on a former occasion; and from reasons similar to those that led us to intrude those Essays on the attention of our readers.

coincide with the orbit, and the[illegible] oscillations are weak continuations of former oscillations.

9. Hence also it is that the centre of the masses of the earth and moon are carried round the sun, by their reciprocal actions and re-actions on and through the medium of space, created by the probable and admitted motion of the sun around the centre of the masses of the solar system; the unequal diffusion and action of the movable fluids in the two hemispheres, causing the earth, at different periods of its orbit, to lengthen and shorten its virtual lever, and to describe an elliptic orbit.

10. The general motions of the earth, as an aggregate, are the sources of all the relative motions which take place upon it; and every motion on the earth is but an appropriation, re-action, or mechanical transfer, of part of the motions of the earth. If the earth were at rest, there could be no motion to transfer; consequently, there could be neither action nor re-action, nor any kind of animal power or loco-motion, nor any aggregate motion or projectile force.

11. All the parts of the earth consolidate and fall towards the centre, because every part is the patient of the rotatory force, which gives them station proportioned to their rarity; and of the paramount orbicular force which impels all the densest masses towards the line of motion; and also because the centre of the earth is the centre of the combined forces or motions of all the united masses.

12. An unattached body, as a stone let fall or projected, returns to the earth, because, at the time when it was unattached or projected, it was the patient of the earth's motions, and the force which raised it ceases to act when it was let fall, or is soon imparted to the air; and because the common force which revolves the earth and atmosphere cannot revolve a stone in the circle in which it revolves the air. In every stratum of the terrestrial mass, the density, multiplied by the velocity of rotation, is, or ought to be, equal; and, if unequal, then bodies rise or fall accordingly: and hence in air a balloon rises, a bubble swims, and a stone falls.

13. As it is with the earth, so it is with all the planetary bodies; they swim in the medium of space, surrounded by atmospheres, which fine off like the down on the seeds of thistles: they are, consequently, moved by very delicate impulses, are turned on their axes by slight combinations, and easily act upon and receive the re-action of their satellites. Like ships in motion, they impart their impulses to bodies in contact with them; and those bodies become, in consequence, the patients of all their motions, while every part, in its own re-actions, necessarily respects the common centre of the motions of the aggregate. Comets move in very eccentric orbits, because they do not move in the plane of the sun's motions or impulses, which is nearly that of the less eccentric planets.

14. When percussion or collision does not produce an equal quantity of aggregate motion in a proportionate change of place in the aggregate; or when the motion received cannot be transferred by diffusion, as when a piece of iron, laid on an anvil, receives the motion of a hammer, or when two pieces of wood are rubbed together, an intestine re-action of the atoms in the iron and wood takes place, accompanied by the perception of heat, and, by a series of phenomena depending on the quantity of motion thus concentrated, and on the acceleration of the same by reiterated blows, rubbings, or transfers of motion.

15. This intestine motion produces various phenomena of the several component atoms of the affected body in regard to one another, and to the heterogeneous media in which they are situated: thus, one quantity creates a perception of heat; another sensibly imparts that perception to the atoms of the surrounding media; another converts the fixed mass into fluids; an acceleration converts the fluids into diverging gas; and a further acceleration, which exceeds the radiating powers of the surrounding media, decomposes those media, exhibiting flame and intense heat, in the solidification of the oxygenous part of the media, and, producing subtle radiations on the rare medium which fills space, thereby affecting the nerves of the eye, imbued with that medium with the perceptions of light.

16. The parting with each degree of atomic motion produces a contrary series of phenomena: thus gas, on parting with its heat or atomic motion to other bodies, becomes fluid; and fluids, by parting with their heat or excited motion, become solids; and the diffusion of heat or atomic motion on such re-conversion, is sensible, when the oxygenous part of atmospheric gas, solidified by respiration, gives out what is called animal heat; and when the same, solidified by combustion, or reduced in volume by compression, gives out heat, and excites the pulsations of light.

17. Resistance is a phenomenon of parting with received motion. A body said to be resisted, is merely parting with its motion to the atoms which it encounters in the media within which it moves; and, as it continues to part with its motion to the radiating atoms, its gradually-diminished energy of motion is, in vulgar language, said to be destroyed by resistance.

18. Friction, like resistance, is a mere phenomenon of parting with motion, but to a fixed body instead of a fluid; and, being a variation of percussion, or transfer of motion without change of place, it produces similar phenomena of intestine atomic motion or heat, which, when continued or accelerated, produces all the other phenomena of accelerated atomic motion or heat.

19. Crystallization is a mere effect of parting with atomic motion, in certain connexions with, or relations to, the atoms of the surrounding media.

20. The phenomena of electricity, galvanism, &c. consist in separations or mechanical decompositions of the component gazeous atoms of plates of electrics, connected and condensed on their opposed surfaces by surfaces of non-electrics: the re-union of which separated strata through a single point of conduit, produces intense phenomena of atomic motion. Thus, glass is coated by tin-foil, air by metal conductors, the atmosphere by clouds and earth, and acids in galvanism by metallic plates; and the electric or galvanic power is within the intervening electrics, or on their surfaces.

21. Loose light bodies placed on the surface of an electrified stratum of coated air, present nearer surfaces to the oppositely-affected surface; and the bodies, being light, are patients of the force exerted within the stratum to restore the disturbed equilibrium of its surfaces; and therefore, by the energy exerted on their surfaces, they are alternately wafted between the affected surfaces of the stratum, creating phenomena which, in the language of the mystical philosophy, are called attraction and repulsion. The power of all affected strata is inversely as the least distance at which the equilibrium of the surfaces will not be restored; and the galvanic series is merely a mechanical means of accumulating or accelerating an original excitement.

22. Chemical affinity affords proof that atoms are compounded in different forms, which coalesce and dove-tail together with more or less facility.

23. Definite sizes in the vegetable and animal kingdoms result from the fixed ratios of the law of increase and decrease, or of accretion and dispersion; which fixed ratios generate a degree of increase, whose limits are determined by the simultaneously acting law of decrease.

24. All the changes visible on the surface of the earth are consequences of volcanoes, terrene or submarine; or of the slow mechanical action of air and water; and the great changes caused by water arise from the successive transfers of the ocean into either hemisphere, by the revolution of the perihelion point of the earth's orbit through the ecliptic in every 20,900 years: the existing strata of organic remains seeming to prove that at least three such revolutions have taken place since the planet of the earth existed in its present form.

25. In fine, Motions of matter subject to regular mechanical laws, acting absolutely or subordinately, generally or locally, on aggregates or atoms, and producing various densities and different degrees of loco-motion and affinity in atoms of matter of different constituent forms, *are the proximate causes of all phenomena;* and, as one series of phenomena depends on another, so all existing phenomena are, in regard to others, physically fit, compatible, and harmonious; and, as matter cannot originate its own motion, so, in considering motion as the proximate cause of all phenomena, we arrive, through the ascending series, at the necessary and sublime FIRST CAUSE *of all motion and all phenomena.*

### REPORT OF DISEASES.

OUR Report for the month just elapsed would, if given in detail, be but little else than an echo of the one preceding it. The prevalent diseases have been similar, only yet more *rife* (to use a neglected English word) amongst the people. Pulmonary inflammation and rheumatism have been more frequent than many practitioners long established in London state they have ever witnessed. The weather has, indeed, been that which is most powerful in producing them,—intense cold with moisture. Last year, at this period, when the season was warm, we had occasion to notice the extreme frequency of apoplexy amongst old persons: it is now a rare disease. Does the same constitutional disposition, differently acted on by external agents, in one season give rise to apoplexy, in another to pneumonia?

There is a fashion in the practice of medicine, as well as in most other human actions; or what is it that makes a man, almost the instant he sees a patient of pulmonic inflammation, call for pen and paper, and write V.S. ad ——, or take out his lancet-case? Venesection, we are persuaded, is far too much employed in pulmonary diseases in London at the present time; that is, it is used too *indiscriminately* by the great mass of practitioners, to the neglect of cupping, leeches, &c. and revulsive measures. But, in stating this, we must not be mistaken: it is in inflammation of the mucous membranes and cellular texture of the lungs that general blood-letting is too extensively employed; it is not at all probable that injury is effected in this way in idiopathic pleurisy, whether affecting the pulmonary or costal portions of the pleura.

From the forcible testimony given by Professor HUFELAND, in a late Number of his Journal, of the efficacy of alkalies in cases of inflammation of the mucous membranes of the trachea and bronchia, after appropriate blood-letting, &c. added to that of a great number of experienced physicians in Germany, (where it is becoming much employed,) we have been induced to inculcate the use of that remedy, in converse with our professional brethren; and we have received, in return, numerous replies indicative of the satisfaction with which they have employed it. It is especially beneficial in children; and, in the state of croup verging to convalescence, it hastens the recovery in a remarkable manner. The carbonate of potash has, for some years past, been a good deal used in hooping-cough, but not in inflammation of the mucous membrane of the respiratory organs generally.

### METEOROLOGICAL JOURNAL.

By Messrs. WILLIAM HARRIS and Co. 50, Holborn, London.

*From December* 20 *to January* 19, *inclusive.*

| Day of Month. | Moon. | Rain gauge | THERM. | | | BAROM. | | De Luc's HYGROM. Dry | Damp | WIND. | | ATMOSPHERIC VARIATION. | | |
|---|---|---|---|---|---|---|---|---|---|---|---|---|---|---|
| Dec | | | | | | | | | | | | | | |
| 20 | | ·07 | 55 | 57 | 47 | 29·68 | 29·80 | 28 | 18 | WSW | W | Rain | Cloud. | Sho'ry |
| 21 | | | 48 | 51 | 51 | 30·00 | 29·75 | 13 | 16 | N | WSW | Cloud. | | |
| 22 | | | 53 | 54 | 44 | 29·74 | 29·6[illegible] | 20 | 20 | W | WNW | Fine | Fine | Cloud. |
| 23 | ☾ | | 45 | 46 | 36 | 29·33 | 29·43 | 15 | 10 | NW | W | Fine | | |
| 24 | | | 39 | 42 | 32 | 29·40 | 29·38 | 7 | 5 | WSW | NNE | Cloud. | Fine | Fine |
| 25 | | | 36 | 40 | 29 | 29·43 | 29·50 | 7 | 6 | NNE | NNW | Fine | Cloud. | Fine |
| 26 | | | 32 | 34 | 31 | 29·55 | 29·53 | 7 | 8 | W | SE | Fine | Cloud. | Foggy |
| 27 | | | 33 | 34 | 31 | 29·50 | 29·52 | 9 | 8 | E | ESE | Fine | Cloud. | Cloud. |
| 28 | | | 35 | 35 | 28 | 29·53 | 29·57 | 10 | 11 | ESE | ENE | Fine | Snow | Cloud. |
| 29 | | | 30 | 31 | 26 | 29·70 | 29·65 | 10 | 9 | N | W | Fine | | |
| 30 | | | 29 | 32 | 30 | 29·55 | 29·45 | 10 | 9 | W | WSW | Cloud. | Fine | Cloud. |
| 31 | ○ | | 31 | 33 | 23 | 29·40 | 29·41 | 10 | 10 | SSW | ESE | Fine | Cloud. | Fine |
| Jan. | | | | | | | | | | | | | | |
| 1 | | | 25 | 27 | 25 | 29·50 | 29·68 | 9 | 10 | E | W | Foggy | Foggy | Fine |
| 2 | | | 31 | 36 | 32 | 29·60 | 29·41 | 13 | 18 | SW | SSW | Cloud. | Fine | Cloud |
| 3 | | | 35 | 37 | 28 | 29·61 | 30·05 | 17 | 15 | N | WNW | Snow | Cloud. | Cloud. |
| 4 | | | 30 | 33 | 22 | 30·10 | 30·11 | 13 | 12 | W | W | Foggy | | |
| 5 | | | 24 | 32 | 27 | 30.13 | 30·17 | 11 | 13 | W | WNW | Foggy | Cloud. | Cloud. |
| 6 | | | 32 | 33 | 33 | 30·14 | 30·20 | 13 | 20 | W | NE | Foggy | Cloud. | Sleet |
| 7 | | | 34 | 34 | 25 | 30·30 | 30.50 | 15 | 12 | E | SE | Cloud. | Fine | Fine |
| 8 | ☽ | | 28 | 29 | 23 | 30·58 | 30·66 | 10 | 11 | E | NE | Fine | Fine | Sno w |
| 9 | | | 27 | 30 | 23 | 30·70 | 30·40 | 11 | 13 | NE | N | Snow | Fine | Sno w |
| 10 | | | 26 | 28 | 26 | 30·45 | 30·20 | 11 | 13 | NE | E | Cloud. | Snow | Fine |
| 11 | | | 29 | 31 | 26 | 30·00 | 29·75 | 14 | 17 | SW | NE | Snow | Snow | Cloud. |
| 12 | | | 27 | 28 | 21 | 30·10 | 30·22 | 12 | 11 | E | ENE | Cloud. | Snow | Fine |
| 13 | | | 24 | 28 | 24 | 30·18 | 30·22 | 10 | 12 | NE | NE | Fine | Snow | Snow |
| 14 | | | 26 | 28 | 15 | 30·24 | 30·04 | 11 | 10 | E | NE | Snow | Fine | Cloud. |
| 15 | ● | | 19 | 21 | 25 | 29·75 | 29·74 | 9 | 14 | SE | N | Fine | Cloud. | Foggy |
| 16 | | | 27 | 29 | 25 | 29·72 | 29·80 | 16 | 15 | NNW | W | Fine | Fine | Foggy |
| 17 | | | 29 | 34 | 29 | 29·71 | 29·52 | 18 | 16 | W | ESE | Cloud. | Cloud. | Fine |
| 18 | | | 31 | 35 | 37 | 29·50 | 29·10 | 17 | 27 | ENE | SW | Snow | Fine | Rain |
| 19 | | | 47 | 49 | 32 | 29·00 | 29·14 | 32 | 24 | WSW | NNE | Cloud. | Cloud. | Fine |

Rain-gauge frozen.

